# Incorporating Information of microRNAs into Pathway Analysis in a Genome-Wide Association Study of Bipolar Disorder

**DOI:** 10.3389/fgene.2012.00293

**Published:** 2012-12-18

**Authors:** Wei-Liang Shih, Chung-Feng Kao, Li-Chung Chuang, Po-Hsiu Kuo

**Affiliations:** ^1^Department of Public Health and Institute of Epidemiology and Preventive Medicine, College of Public Health, National Taiwan UniversityTaipei, Taiwan; ^2^Infectious Diseases Research and Education Center, Department of Health – Executive Yuan and National Taiwan UniversityTaipei, Taiwan; ^3^Research Center for Genes, Environment and Human Health, National Taiwan UniversityTaipei, Taiwan; ^4^Department of Nursing, Cardinal Tien College of Healthcare and ManagementYilan, Taiwan

**Keywords:** microRNA, bipolar disorder, pathway analysis, genome-wide association, ion channel

## Abstract

MicroRNAs (miRNAs) are known to be important post-transcriptional regulators that are involved in the etiology of complex psychiatric traits. The present study aimed to incorporate miRNAs information into pathway analysis using a genome-wide association dataset to identify relevant biological pathways for bipolar disorder (BPD). We selected psychiatric- and neurological-associated miRNAs (*N* = 157) from PhenomiR database. The miRNA target genes (miTG) predictions were obtained from microRNA.org. Canonical pathways (*N* = 4,051) were downloaded from the Molecule Signature Database. We employed a novel weighting scheme for miTGs in pathway analysis using methods of gene set enrichment analysis and sum-statistic. Under four statistical scenarios, 38 significantly enriched pathways (*P*-value < 0.01 after multiple testing correction) were identified for the risk of developing BPD, including pathways of ion channels associated (e.g., gated channel activity, ion transmembrane transporter activity, and ion channel activity) and nervous related biological processes (e.g., nervous system development, cytoskeleton, and neuroactive ligand receptor interaction). Among them, 19 were identified only when the weighting scheme was applied. Many miRNA-targeted genes were functionally related to ion channels, collagen, and axonal growth and guidance that have been suggested to be associated with BPD previously. Some of these genes are linked to the regulation of miRNA machinery in the literature. Our findings provide support for the potential involvement of miRNAs in the psychopathology of BPD. Further investigations to elucidate the functions and mechanisms of identified candidate pathways are needed.

## Introduction

Bipolar disorder (BPD) is a highly heritable psychiatric illness. The genetic components were estimated to account for as high as ∼80% of phenotypic variability (McGuffin et al., 2003). Although many candidate and genome-wide association (GWA) studies have conducted to investigate the complex nature of pathogenetics in BPD, previously reported genetic findings only account for a small proportion of its heritability (Gershon et al., 2011). The missing heritability may be partially explained by the limited numbers, types, and frequency of susceptible variants that currently genotyped in high-throughput array, and other mechanisms such as gene × gene or gene × environment interactions, as well as the heterogeneity in phenotype definitions across studies (Manolio et al., 2009). Nevertheless, large-scale GWA studies remain to be an efficient and promising study design to uncover the underlying etiology of complex psychiatric disorders (Sullivan and Investigators, 2012), while new theoretical framework and statistical approaches must be taken into consideration.

Recently, pathway-based analysis, which simultaneously tests a group of functionally related genes, has been widely used as an alternative and complementary strategy to bring more insights into the biological mechanisms of disease of interest (Wang et al., 2010). In addition, inclusion of prior information from other aspects, such as gene expression or gene regulation in GWAS analysis offers great opportunities to identify new association findings and to generate novel hypotheses (Tintle et al., 2009a). For instance, two prior GWA studies in type 2 diabetes and osteoporosis applied integrative approaches that used gene expression data and pathway-based analysis to identify novel associated pathways and loci (Hsu et al., 2010; Zhong et al., 2010). In addition to gene expression information, other types of data could also be incorporated into pathway-based analysis using GWA data, such as methylation (Chuang et al., 2012) and microRNAs (miRNAs) patterns, especially disease-associated miRNAs.

The miRNAs are one kind of functional non-coding RNAs acting as post-transcriptional regulators for translation and the stability of mRNAs, which involved in a wide range of biological processes, including regulation of brain and neuronal development (Fiore et al., 2008). The miRNA dysregulation has been reported to play important roles in the etiology of many diseases, including complex psychiatric traits (Xu et al., 2010). Previously, many psychiatric- and neurological-associated miRNAs were identified from expression studies of postmortem brain and animal models (Forero et al., 2010), and from genetic association studies of variants in genes encoding miRNAs and binding site of miRNAs target genes (Muinos-Gimeno et al., 2009; The Schizophrenia Psychiatric Genome-Wide Association Study (GWAS) Consortium, 2011).

We have known that an individual miRNA could target hundreds of mRNA molecules (Lim et al., 2005), therefore the target genes of a phenotype-related miRNA may potentially associate with the trait. For example, abnormal expression level of brain-expressed miR-132 has been reported to be associated with psychiatric disorders via affecting the expression of brain-derived neurotrophic factor that is involved in dendritic plasticity (Klein et al., 2007; Wayman et al., 2008; Askland et al., 2009; Forero et al., 2010). In addition, different psychiatric disorders (e.g., schizophrenia and BPD) may share some degree of the genetic factors through the involvement of similar biological pathways. Thus, a single miRNA may play roles in the etiology of more than one psychiatric disorder. Given these features of miRNAs, it is our interest to incorporate information of phenotype-related miRNAs and their predicted targets into the GWA analysis, which provide a new avenue for researchers to investigate the underlying genetic components that are associated with BPD.

In the current study, we performed pathway-based analysis using large-scale GWA dataset of BPD in combination with the data source of miRNAs. The psychiatric- and neurological-associated miRNAs were identified from PhenomiR database and miRNA target predictions were obtained from microRNA.org database. Our main goal is to better identify genes and important biological pathways to be associated with BPD while incorporating the regulatory information of miRNAs.

## Materials and Methods

**Figure 1:**
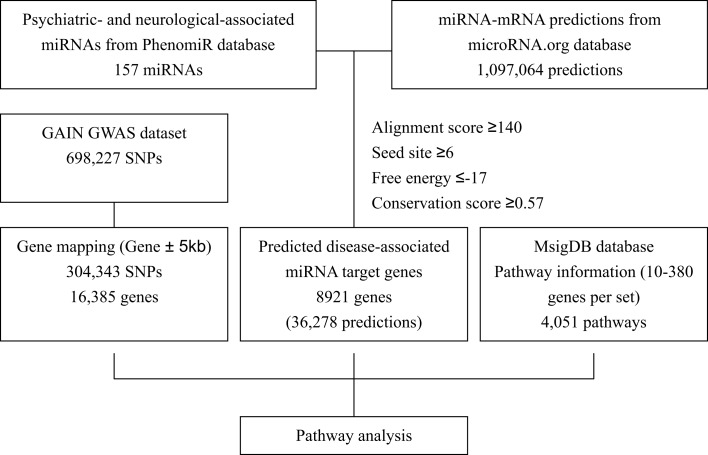
**The flowchart of identification of psychiatric- and neurological-associated miRNAs and their target genes and information for pathway analysis**.

Figure 1 shows the data integration flowchart in our study to identify the psychiatric- and neurological-associated miRNAs and their target genes, and the annotated pathways for pathway-based analysis. Details are mentioned below.

### Genome-wide association dataset

The Genetic Association Information Network (GAIN) GWA dataset was downloaded from dbGaP^1^. We extracted BPD dataset from the GAIN: full details of subject enrollment and genotyping can be obtained in the original article (The GAIN Collaborative Research Group, 2007). The GWA dataset of BPD comprised 1,001 BPD cases and 1,034 controls, which used Affymetrix Genome-Wide Human SNP Array 6.0 platform for SNP (single nucleotide polymorphism) genotyping. After applying quality control filters and excluding SNPs in sexual chromosomes, there were 698,227 autosomal SNPs in the GWA dataset in our analyses.

### Identification of target genes of psychiatric disease-associated miRNAs

Information of disease-associated miRNAs was downloaded from the PhenomiR database (Ruepp et al., 2010). The PhenomiR collected published miRNA-disease associations via manual curation. It annotates diseases into 22 classes according to the Online Mendelian Inheritance in Man (OMIM) Morbid Map. In our analysis, we selected all miRNAs that are associated with neurological and psychiatric classes as the candidates of disease-associated miRNAs. In total, there were 157 unique miRNAs to form 293 miRNA-disease association pairs.

The miRNA target predictions were obtained from micorRNA.org database^2^ (Betel et al., 2008). The micorRNA.org performed miRNA target prediction using miRanda algorithm (John et al., 2004) and scored the likelihood of mRNA downregulation of predicted target sites by using mirSVR algorithm (Betel et al., 2010). The combination of miRanda-mirSVR approach has been shown to effectively identify target predictions to cover a significant number of non-canonical sites, and has competitive ability in predicting expression changes of mRNA or proteins when comparing with other target prediction methods (Betel et al., 2010). In total, 1,097,064 “good mirSVR score-conserved miRNA” target predictions were used in this procedure. Combining these two datasets while follows the criteria of alignment score ≥140, seed site ≥6, free energy ≤−17, and conservation score ≥0.57 (Figure 1), we identified 8,921 genes which were predicted to be the targets of psychiatric- and neurological-associated miRNAs.

We also used another miRNA target prediction algorithm, DIANA-microT, which considers not only strong binding (at least seven consecutive Watson-Crick base pairing nucleotides) but also weak binding ability (only six paired nucleotides or G:U wobble pairs) to predict the miRNA target genes (miTG; Maragkakis et al., 2009a,b). DIANA-microT provides scores for miTG as an indicator for the probability of being a real target site. The calculation of an overall miTG score mainly based on scoring all binding types and conservation profile of all putative miRNA recognition element (MRE) within the 3′UTR using the weighted sum method. Therefore, target genes of psychiatric- and neurological-associated miRNAs with high predictive probability in significant pathways were filtered by the DIANA-microT algorithm. We used a miTG score greater than 19 (a strict threshold) as the selection criterion, which implicates the predicted target was highly reliable being a true miRNA target.

### Statistical analysis

We used PLINK (version 1.07) to conduct single marker association analyses with additive model (Purcell et al., 2007). We first mapped SNPs to genes to obtain gene-level statistic for BPD using the GWA dataset in GAIN. SNPs were mapped to genes if they located within 5 kb of the 5′ upstream and 3′ downstream of a gene using NCBI human genome build 36. For each gene, the smallest *P*-value (*P*_min_) among all SNPs within the gene region was used to represent the gene-level statistic. In total, there were 304,343 SNPs assigned into 16,385 genes in the GWA dataset of BPD.

Annotated pathways were obtained from the Molecule Signature Database, MsigDB (Subramanian et al., 2005). MsigDB consists of several online pathway databases, including Kyoto Encyclopedia of Genes and Genomes (KEGG), BioCarta, Reactome, Gene Ontology (GO) terms, and gene sets collected from published literature. From MsigDB, we obtained 4,726 pathways that cover 22,429 genes. Pathways containing less than 10 genes or more than 380 genes were excluded to avoid bias due to extreme small or large pathway size. Thus, there were 4,051 canonical pathways in the pathway-based analyses using the GWA dataset of BPD in the present study.

Pathway-based analyses were conducted using both competitive and self-contained approaches (Wang et al., 2010) to capture a broader range of important pathways. The gene set enrichment analysis (GSEA), as a competitive method, first ranks *P*_min_ values of all genes from the smallest to the largest. Then, for a given pathway, an enrichment score (ES) was calculated based on gene-wise statistic values (*t*_j_), which were defined as the χ^2^ statistic of the corresponding most significant SNP to evaluate association signals (Wang et al., 2007). The sum-statistic (SUM) approach, as a self-contained test, sums up gene-wise statistic values (*t*_*i*_) over the set of genes $\left(\Sigma_{i=1}^{S}t_{i}\right)$ in a specific pathway (Tintle et al., 2009b). The details of calculation procedures were provided in our previous study (Kao et al., 2012).

### Weighting procedure

We employed a weighting scheme for genes that were predicted to be psychiatric- and neurological-associated miRNA targets in every annotated pathway. First, we calculated the overall proportion of SNPs with *P*-value <0.05 in the whole GAIN dataset. Second, in a given gene, the proportion of significant SNPs was calculated and then compared with the proportion of significant SNPs in the whole GWA dataset to evaluate whether this gene was informative. The detail of weighting procedures was described as below. For each pathway, *n* and *m* represent the number of miTGs and non- miTGs, respectively. *K*_n_ and *K*_m_ were the number of informative genes in the n miTGs and m non-miTGs, respectively. Therefore, the proportion of informative genes in the miTGs and non-miTGs were *K*_n_/n and *K*_m_/m. The Harmonic average (H), defined as 1/[(1/m*K*_n_) + (1/n*K*_m_)], was used as the basis of our weighting scheme for calculating the gene-wise weights of the miRNA and non-miTGs in a pathway and to minimize the potential bias in the pathway analysis due to the variation of pathway size. If *K*_n_/n was greater than *K*_m_/m, the weights for miTGs and non-miTGs were assigned mk_n_/H and nk_m_/H, respectively. If no informative genes exist in non-miRNA genes, a weight one was assigned to non-miRNA genes; while a weight, ranging from one to six, according to the proportion of informative genes (using 0.1, 0.3, 0.5, 0.7, and 0.9 as cut-off values), was assigned to miRNA genes. Otherwise, equal weights were used for the two sets of genes.

For each pathway, weights were assigned to miTGs and non-miTGs. Then all genes were classified into S set and NS set according to their involvement in a pathway or not. When using competitive methods, genes within a pathway were compared with genes not within the pathway. Regarding to self-contained methods, only genes within the pathway were considered. A total of 5,000 permutations were performed to evaluate the empirical significance level of each pathway. To account for multiple testing issues in the analyses, algorithm proposed by Benjamini and Hochberg (1995) was used to control for false discovery rate (FDR).

## Results

A total of 4,051 pathways were constructed and tested for associations with the risk of BPD using the GWA dataset of BPD. With the inclusion of 157 psychiatric- and neurological-associated miRNAs as the prior information into pathway-based analyses, we identified many enriched pathways for BPD. Under four testing scenarios, including weighted and non-weighted GSEA and SUM statistics, there were more than 100 significant pathways associated with BPD at the level of empirical *P*-value < 0.01, and the number was reduced to more than 40 after FDR correction (Table A1 in Appendix). Comparing with non-weighting scenario, pathway analysis under the weighting scenario identified additional 20 and 223 significant pathways (FDR < 0.01) by using GSEA and SUM methods, respectively (Table A2 in Appendix). Under the non-weighted scheme, the number of enriched pathways identified by both the GSEA and SUM methods with FDR < 0.01 was 43, while the number was 38 under the weighted scheme. The union set of these enriched pathways were in total 62 pathways (Table A3 in Appendix), including 18 annotated GO, 7 KEGG, and 37 curated gene sets. Among these pathways, 19 significant pathways were identified by both the GSEA and SUM methods using both non-weighted and weighted scheme.

Table 1 showed 19 enriched pathways with stringent criterion of FDR < 0.01, including four GO gene sets and 15 curated gene sets, which exhibited strong associations with BPD under all four statistical scenarios. The three significant GO gene sets, cation transmembrane transporter activity, gated channel activity, and ion transmembrane transporter activity, were ion channel/transporter related. The fourth GO gene set was nervous system development, which was reported to be associated with BPD previously. After performing our weighting scheme, 19 additional pathways were identified at the significance level of FDR < 0.01 (Table 2), including six annotated GO, 3 KEGG, and 10 curated gene sets. Many of them are novel findings for BPD, such as cytoskeleton, retinol metabolism, drug metabolism other enzymes, etc. In total, we found 38 significant enriched pathways for BPD.

**Table 1:** **Enriched pathways with FDR_BH_ < 0.01 under weighted and non-weighted scenarios using GSEA and SUM methods**.

Pathway	Type	No. of genes in pathway	No. of miRNA target genes	No. of non-miRNA target genes	% of miRNA target genes*	GSEA				SUM			
						Non-weighted		Weighted		Non-weighted		Weighted	
						Empirical *P*-value	FDR_BH_	Empirical *P*-value	FDR_BH_	Empirical *P*-value	FDR_BH_	Empirical *P*-value	FDR_BH_
Cation transmembrane transporter activity	GO	211	109	85	56.2	<2e−4	<2e−4	<2e−4	<2e−4	<2e−4	<2e−4	<2e−4	<2e−4
Gated channel activity	GO	121	55	59	48.2	<2e−4	<2e−4	<2e−4	<2e−4	<2e−4	<2e−4	<2e−4	<2e−4
Ion transmembrane transporter activity	GO	275	128	123	51.0	<2e−4	<2e−4	<2e−4	<2e−4	<2e−4	<2e−4	<2e−4	<2e−4
Nervous system development	GO	382	191	135	58.6	<2e−4	<2e−4	<2e−4	<2e−4	<2e−4	<2e−4	<2e−4	<2e−4
Acevedo liver cancer with H3K27ME3 up	Curated	295	109	105	50.9	<2e−4	<2e−4	<2e−4	<2e−4	<2e−4	<2e−4	<2e−4	<2e−4
Acevedo liver cancer with H3K9ME3 up	Curated	141	50	55	47.6	<2e−4	<2e−4	<2e−4	<2e−4	<2e−4	<2e−4	<2e−4	<2e−4
Bertucci medullary Vs. ductal breast cancer dn	Curated	177	88	54	62.0	<2e−4	<2e−4	<2e−4	<2e−4	<2e−4	<2e−4	<2e−4	<2e−4
Dacosta UV response Via. ERCC3 TTD DN	Curated	76	50	19	72.5	<2e−4	<2e−4	<2e−4	<2e−4	<2e−4	<2e−4	<2e−4	<2e−4
HAMAI apoptosis VIA trail UP	Curated	334	176	134	56.8	<2e−4	<2e−4	<2e−4	<2e−4	<2e−4	<2e−4	<2e−4	<2e−4
Lindgren bladder cancer cluster 3 DN	Curated	223	98	86	53.3	<2e−4	<2e−4	<2e−4	<2e−4	<2e−4	<2e−4	<2e−4	<2e−4
Manalo hypoxia UP	Curated	211	123	58	68.0	<2e−4	<2e−4	<2e−4	<2e−4	<2e−4	<2e−4	<2e−4	<2e−4
Martinez response to trabectedin	Curated	42	26	13	66.7	<2e−4	<2e−4	<2e−4	<2e−4	<2e−4	<2e−4	<2e−4	<2e−4
RIGGI ewing sarcoma progenitor UP	Curated	429	222	133	62.5	<2e−4	<2e−4	<2e−4	<2e−4	<2e−4	<2e−4	<2e−4	<2e−4
Siligan bound by EWS FLT fusion	Curated	36	18	16	52.9	<2e−4	<2e−4	<2e−4	<2e−4	<2e−4	<2e−4	<2e−4	<2e−4
Vecchi gastric cancer early DN	Curated	394	161	125	56.3	<2e−4	<2e−4	<2e−4	<2e−4	<2e−4	<2e−4	<2e−4	<2e−4
Verhaak AML with NPM1 mutated DN	Curated	266	130	93	58.3	<2e−4	<2e−4	<2e−4	<2e−4	<2e−4	<2e−4	<2e−4	<2e−4
Wang SMARCE1 targets UP	Curated	170	92	52	63.9	<2e−4	<2e−4	<2e−4	<2e−4	<2e−4	<2e−4	<2e−4	<2e−4
Onder CDH1 signaling via CTNNB1	Curated	85	45	31	59.2	<2e−4	<2e−4	<2e−4	<2e−4	<2e−4	<2e−4	<2e−4	0.003
Sabates colorectal adenoma DN	Curated	292	118	112	51.3	<2e−4	<2e−4	<2e−4	<2e−4	<2e−4	0.006	<2e−4	<2e−4

*Proportion of miRNA target genes was obtained by calculating the number of miRNA target genes divided by the total number of miRNA target genes plus non-miRNA target genes.

*GO, Gene Ontology; KEGG, Kyoto Encyclopedia of Genes and Genomes; GSEA, gene set enrichment analysis; SUM, sum-statistic approach; FDR_BH_, correct for false discovery rates using Benjamini & Hochberg’s method*.

*Empirical *P*-value and FDR_BH_ “<2e−4” meant that no one had greater score than the actual score among the 5,000 permutations for the analysis of each pathway*.

**Table 2:** **Enriched pathways with FDR_BH_<0.01 level under weighting scheme using both GSEA and SUM methods**.

Pathway	Type	No. of genes in pathway	No. of miRNA target genes	No. of non-miRNA target genes	% of miRNA target genes*	GSEA				SUM			
						Non-weighted		Weighted		Non-weighted		Weighted	
						Empirical *P*-value	FDR_BH_	Empirical *P*-value	FDR_BH_	Empirical *P*-value	FDR_BH_	Empirical *P*-value	FDR_BH_
Synaptic transmission	GO	172	77	81	48.7	**<2e-4**	0.021	**<2e-4**	**<2e-4**	**<2e-4**	**0.006**	**<2e-4**	**<2e-4**
Neurological system process	GO	377	139	202	40.8	**<2e-4**	0.021	**<2e-4**	**<2e-4**	**<2e-4**	0.021	**0.001**	**<2e-4**
Ion channel activity	GO	147	70	69	50.4	**0.001**	0.036	**<2e-4**	**<2e-4**	**<2e-4**	**<2e-4**	**<2e-4**	**<2e-4**
Substrate specific channel activity	GO	154	74	72	50.7	**0.001**	0.041	**<2e-4**	**<2e-4**	**<2e-4**	**<2e-4**	**<2e-4**	**<2e-4**
Transmission of nerve impulse	GO	187	83	90	48.0	**0.001**	0.041	**<2e-4**	**<2e-4**	**<2e-4**	0.018	**0.001**	**<2e-4**
Cytoskeleton	GO	361	173	150	53.6	0.030	0.285	**<2e-4**	**<2e-4**	**<2e-4**	0.252	0.042	**<2e-4**
Neuroactive ligand receptor interaction	KEGG	272	94	144	39.5	**<2e-4**	0.021	**<2e-4**	**<2e-4**	**<2e-4**	0.123	0.012	**<2e-4**
Retinol metabolism	KEGG	64	19	38	33.3	**0.001**	0.029	**<2e-4**	**<2e-4**	**<2e-4**	0.215	0.032	**<2e-4**
Drug metabolism other enzymes	KEGG	51	20	24	45.5	**0.002**	0.057	**<2e-4**	**<2e-4**	**<2e-4**	0.425	0.105	**<2e-4**
Hatada methylated in lung cancer up	Curated	367	161	144	52.8	**<2e-4**	**<2e-4**	**<2e-4**	**<2e-4**	**0.001**	0.014	**<2e-4**	**<2e-4**
Delys thyroid cancer DN	Curated	214	109	86	55.9	**<2e-4**	0.013	**<2e-4**	**<2e-4**	**<2e-4**	**<2e-4**	**<2e-4**	**<2e-4**
Horiuchi WTAP targets up	Curated	323	147	119	55.3	**<2e-4**	0.013	**<2e-4**	**<2e-4**	**<2e-4**	**<2e-4**	**<2e-4**	**<2e-4**
Thum systolic heart failure DN	Curated	248	136	61	69.0	**0.001**	0.029	**<2e-4**	**<2e-4**	**<2e-4**	**<2e-4**	**<2e-4**	**<2e-4**
Iwanaga carcinogenesis by KRAS Pten DN	Curated	445	161	119	57.5	**0.001**	0.036	**<2e-4**	**<2e-4**	**<2e-4**	**<2e-4**	**<2e-4**	**<2e-4**
Doane response to androgen DN	Curated	248	119	94	55.9	**0.006**	0.116	**<2e-4**	**<2e-4**	**0.001**	0.021	**<2e-4**	**<2e-4**
Foster inflammatory response LPS DN	Curated	486	219	122	64.2	0.017	0.205	**<2e-4**	**<2e-4**	**<2e-4**	**0.006**	**<2e-4**	**<2e-4**
Acevedo liver cancer with H3K27ME3 DN	Curated	226	73	87	45.6	0.019	0.216	**<2e-4**	**<2e-4**	0.210	0.615	**<2e-4**	**<2e-4**
Boylan multiple myeloma C D DN	Curated	328	100	113	46.9	0.020	0.224	**<2e-4**	**<2e-4**	0.027	0.196	**0.001**	**0.009**
Yauch hedgehog signaling paracrine DN	Curated	385	104	108	49.1	0.058	0.396	**<2e-4**	**<2e-4**	**<2e-4**	**0.006**	**<2e-4**	**<2e-4**

*Proportion of miRNA target genes was obtained by calculating the number of miRNA target genes divided by the total number of miRNA target genes plus non-miRNA target genes.

*Empirical *P*-value or FDR_BH_ with values less than 0.01 were shown in bold*.

*GO, Gene Ontology; KEGG, Kyoto Encyclopedia of Genes and Genomes; GSEA, gene set enrichment analysis; SUM, sum-statistic approach; FDR_BH_, correct for false discovery rates using Benjamini & Hochberg’s method*.

*Empirical *P*-value and FDR_BH_ “<2e−4” meant that no one had greater score than the actual score among the 5,000 permutations for the analysis of each pathway*.

### miRNA target prediction

The results of miRNA target predictions in 38 enriched pathways were further examined. Initially, miRNA target prediction was performed by using miRanda-mirSVR approach with previously described filtering criteria (Figure 1). There were 2,438 unique genes in 38 significantly enriched pathways. Among them, 546 had *P*_min_-value less than 0.01. Table 3 summarized the results of miRNA target predictions for these 546 genes. On average, 34.4% of the predicted miTGs had *P*_min_-value less than 0.01 in enriched pathways, indicating a higher probability of showing associations with BPD. As expected, the larger the numbers of genes or the numbers of miTGs in a pathway, the higher number of miRanda-mirSVR prediction was observed (correlation coefficient = 0.74 and 0.82, respectively) in the 38 enriched pathways. We then applied the second prediction algorithm, DIANA-microT, to increase the stringency of target genes prediction. These results were also shown in Table 3. By using the strict threshold at miTG score 19, we filtered out the predictions with less probability of correct prediction. Among the 38 pathways, as high as 88.9% of the miRanda-mirSVR predictions could be also predicted by DIANA-microT (ranged from 36.8 to 88.9%). The numbers of miRNA target predictions were also reduced (from 0 to 28). In total, there were 469 miRNA target predictions with miTG score >19 for genes with *P*_min_-value of target gene <0.01, which consisted of 113 unique genes and 45 miRNAs. Among these predictions, 22 miTGs were involved in more than three enriched pathways. The 22 miTGs and their corresponding associated miRNAs are displayed in Table 4. The functions of these genes are mainly related to potassium and calcium ion channels (e.g., *KCNMA1*, *KCNQ5*, *KCNK2*, *PKD2*, and *RYR3*), collagen (e.g., *COL1A2*, *COL27A1*, and *COL5A1*), and axon guidance (e.g., *NF1B*, *NAV3*, and *PTPRD*).

**Table 3:** **Predictions of psychiatric- and neurological-associated miRNA target genes with *P*_min_ < 0.01 in 38 significantly enriched pathways**.

Pathway	No. of genes in pathway	No. of miRNA target genes	Genes with *P*_min_ < 0.01			
			miRanda-mirSVR prediction			DIANA-microT prediction*
			No. of genes	No. of miRNAs	No. of miRNA:gene predictions	miTG score >19
**ALL PATHWAY FDR_BH_ < 0.01**						
Cation transmembrane transporter activity	211	109	32	49	87	5
Gated channel activity	121	55	20	41	56	5
Ion transmembrane transporter activity	275	128	36	49	100	6
Nervous system development	382	191	63	70	278	28
Acevedo liver cancer with H3K27ME3 up	295	109	42	76	173	26
Acevedo liver cancer with H3K9ME3 up	141	50	19	49	72	7
Bertucci medullary vs. ductal breast cancer DN	177	88	33	64	111	15
Dacosta UV response via ERCC3 TTD DN	76	50	24	56	105	26
Hamai apoptosis via trail up	334	176	41	68	159	9
Lindgren bladder cancer cluster 3 DN	223	98	32	57	117	17
Manalo hypoxia up	211	123	37	65	123	10
Martinez response to trabectedin	42	26	15	38	52	8
Riggi ewing sarcoma progenitor up	429	222	69	83	290	21
Siligan bound by ews FLT1 fusion	36	18	14	47	68	16
Vecchi gastric cancer early DN	394	161	47	69	169	10
Verhaak AML with NPM1 mutated DN	266	130	35	62	119	2
Wang SMARCE1 targets up	170	92	29	54	99	17
Onder CDH1 signaling via CTNNB1	85	45	13	37	47	11
Sabates colorectal adenoma DN	292	118	38	62	130	12
**GSEA-WEIGHTED FDR_BH_ < 0.01 AND SUM-WEIGHTED FDR_BH_ < 0.01**						
Synaptic transmission	172	77	26	61	126	8
Neurological system process	377	139	49	69	225	12
Ion channel activity	147	70	22	42	65	5
Substrate specific channel activity	154	74	22	42	65	5
Transmission of nerve impulse	187	83	28	61	128	8
Cytoskeleton	361	173	36	68	160	7
Neuroactive ligand receptor interaction	272	94	28	58	112	2
Retinol metabolism	64	19	12	11	19	3
Drug metabolism other enzymes	51	20	13	11	20	0
Hatada methylated in lung cancer up	367	161	41	67	120	22
Delys thyroid cancer dn	214	109	42	67	168	20
Horiuchi wtap targets up	323	147	48	73	213	23
Thum systolic heart failure dn	248	136	41	77	192	12
Iwanaga carcinogenesis by kras pten DN	445	161	39	65	133	28
Doane response to androgen dn	248	119	29	53	101	7
Foster inflammatory response LPS DN	486	219	43	73	169	22
Acevedo liver cancer with H3K27ME3 DN	226	73	20	51	102	18
Boylan multiple myeloma C D DN	328	100	23	54	83	8
Yauch hedgehog signaling Paracrine DN	385	104	30	60	123	8

*DIANA-microT algorithm was applied to the predictions obtained by using miRanda-mirSVR approach with miTG score > 19 (the prediction score of a miRNA and its trarget gene calculated by DIANA-microT algorithm).

**Table 4:** **List of miRNA target genes and associated miRNAs in 38 significantly enriched pathways**.

Gene symbol	Gene description	Pathway count	*P*_min_	miRNA					
KCNQ5	Potassium voltage-gated channel, KQT-like subfamily, member 5	8	0.00212	hsa-miR-181c	hsa-miR-181d				
PKD2	Polycystic kidney disease 2	7	0.00765	hsa-miR-106b	hsa-miR-20b				
RYR3	Ryanodine receptor 3	6	0.00038	hsa-miR-124					
CNTN4	Contactin 4	5	0.00065	hsa-miR-148b					
JAG1	Jagged 1	5	0.00020	hsa-miR-26b					
KCNMA1	Potassium large conductance calcium-activated channel, subfamily M, alpha member 1	5	0.00112	hsa-miR-106a	hsa-miR-17	hsa-miR-93			
COL1A2	Collagen, type I, alpha 2	5	0.00316	hsa-let-7b	hsa-miR-29a	hsa-miR-29b	hsa-miR-29c		
COL27A1	Collagen, type XXVII, alpha 1	4	0.00022	hsa-let-7b	hsa-let-7i				
COL5A1	Collagen, type V, alpha 1	4	0.00141	hsa-miR-29a	hsa-miR-29b	hsa-miR-29c			
DLC1	Deleted in liver cancer 1	4	0.00271	hsa-miR-429					
KIF1B	Kinesin family member 1B	4	0.00558	hsa-miR-15a	hsa-miR-497				
ABCA1	ATP-binding cassette, subfamily A (ABC1), member 1	3	0.00908	hsa-miR-17					
ANK2	Ankyrin 2, neuronal	3	0.00029	hsa-miR-106a	hsa-miR-9	hsa-miR-93			
FLRT2	Fibronectin leucine rich transmembrane protein 2	3	0.00395	hsa-miR-101					
JARID2	Jumonji, AT rich interactive domain 2	3	0.00638	hsa-miR-130a					
KCNK2	Potassium channel, subfamily K, member 2	3	0.00773	hsa-miR-27a	hsa-miR-27b				
MYO10	Myosin X	3	0.00697	hsa-miR-124					
NAV3	Neuron navigator 3	3	0.00691	hsa-miR-29a	hsa-miR-29b	hsa-miR-29c			
NFIB	Nuclear factor I/B	3	0.00306	hsa-miR-25	hsa-miR-29b	hsa-miR-363			
PAPPA	Pregnancy-associated plasma protein A, pappalysin 1	3	0.00112	hsa-miR-15a	hsa-miR-15b				
PTPRD	Protein tyrosine phosphatase, receptor type, D	3	0.00148	hsa-let-7b	hsa-let-7g	hsa-let-7i	hsa-miR-106a	hsa-miR-106b	hsa-miR-124
				hsa-miR-133b	hsa-miR-17	hsa-miR-20b	hsa-miR-26b	hsa-miR-93	
ZFHX3	Zinc finger homeobox 3	3	0.00175	hsa-miR-15a	hsa-miR-15b	hsa-miR-195	hsa-miR-27a	hsa-miR-27b	hsa-miR-381

*Only miRNA target genes with *P*_min_ < 0.01 and involved in at least three pathways were listed*.

## Discussion

Analyzing GWA dataset with pathway-based approach utilizes information of multiple loci with similar physiological functions to bring biological insights into the mechanisms of BPD (Torkamani et al., 2008; Askland et al., 2009; Holmans et al., 2009; Peng et al., 2010). Integrating other data sources into the analysis framework further offers more opportunities in identifying disease-associated loci (Wang et al., 2010). The current study especially focuses on information obtained from miRNAs, which are essential in the regulation processes of brain and neuronal development. We performed pathway-based analyses using a GWA dataset of BPD while incorporating the disease-associated miRNA information into analysis. Many important pathways were identified through our analysis framework.

First, four enriched GO terms were identified for BPD, including cation transmembrane transporter activity, gated channel activity, ion transmembrane transporter activity, and nervous system development. Three of them are ion channel/transporter related. Adding the weighting scheme by miRNA information, we further identified two channel-related pathways (Table 2), ion channel activity and substrate specific channel activity. The involvement of ion channels in the etiology of BPD was also implicated in other studies for BPD (Askland et al., 2009). We have known that ion channels and transporters are essential components in regulating neuronal excitability. Abnormality of ion channels has been suggested to be a plausible mechanism underlying BPD. To explain the recurrence and cycling nature of mood episodes in BPD, a kindling model was proposed as these clinical conditions are the consequences of neuronal hyperexcitability, which is linked to abnormal functions of ion channels (Mazza et al., 2007; Blumenfeld et al., 2009). Similarly, results in recent GWA and gene expression studies also support the involvement of ion channel genes in the etiology of BPD (Sklar et al., 2008, 2011; Smolin et al., 2012). Thus, genes in the ion channels or their regulatory loci have been attractive candidates in studying the underlying mechanism for BPD. Our miTGs prediction grants further support for this line of evidence.

In our identified 38 significantly enriched pathways, many miTGs were functionally related to ion channels, especially for potassium (e.g., *KCNMA1*, *KCNQ5*, and *KCNK2*) and calcium (e.g., *RYR3* and *PKD2*) channels. All these genes involved in multiple significant pathways. For instance, *KCNMA1* is a calcium-activated potassium channel and *KCNQ5* belongs to the voltage-gated delayed rectifier potassium channel gene family. Both of them play important roles in the regulation of neuronal excitability (Laumonnier et al., 2006; Brown and Passmore, 2009). Molecular and functional studies found that defects of *KCNMA1* contribute to autism and mental retardation (Laumonnier et al., 2006). For *KCNK2* gene that encodes for a two-pore-domain background potassium channel, a recent genetic study revealed its association with susceptibility of major depressive disorder and response to antidepressant treatment (Liou et al., 2009). Additionally, several susceptible genes that cause abnormality in calcium signaling, such as *CACNA1C* and *Bcl-2*, were reported to be associated with BPD (Sklar et al., 2008, 2011; Distelhorst and Bootman, 2011). *RYR3*, a brain-specific ryanodine receptor for controlling intracellular calcium concentration, was found to be a susceptible gene for schizophrenia (Leonard and Freedman, 2006). In addition, the RYR3 knockout mice exhibited some abnormal behaviors, including hyperlocomoter activity and decreased social interaction (Matsuo et al., 2009). Although how these behaviors defects and functional defects of ion channels link with the pathology of BPD are still unclear, it warrants to conduct further basic and functional research to investigate the roles of ion channels in BPD.

Second, applying the weighting scheme for psychiatric- and neurological-associated miRNAs in the analysis, we identified several significant pathways for BPD that were involved many nervous related biological processes in Table 2 (Li et al., 2005; Nakatani et al., 2006; Ryan et al., 2006; Bremner and McCaffery, 2008; Ramocki and Zoghbi, 2008; Torkamani et al., 2008; Askland et al., 2009). Some pathways are novel findings for BPD but show their biological plausibility to neurological disorders in general, such as retinol metabolism (Maden, 2002), while other pathways are novel findings specific to BPD (not reported in other neurological disorders, such as drug metabolism other enzymes). Of note, it is known that necessity of cytoskeletal modulation play a role in the processes of nervous system development (Ramocki and Zoghbi, 2008). Additionally, neuroactive ligand receptor interaction pathway was reported to be associated with substance addiction, which is commonly observed comorbid condition in BPD patients (Li et al., 2005).

The miRNA regulation potentially contributes to the functions of these associated pathways in BPD. Recent findings exhibited the essential roles of miRNA machinery in many aspects of nervous system, including Dicer and miR-124 in neuronal development and miR-134 in synaptic development (Gao, 2008; Saba and Schratt, 2010). Regulation of calcium channel gene expression by miR-103 was also reported (Favereaux et al., 2011). In addition to enriched GO and KEGG pathways, we also identified many significant pathways that were obtained from curated data in the literature in MsigDB, which was mainly based on gene expression studies related with multiple cancers. Examining the functions of miRNA-associated genes in these enriched pathways suggested the involvement of different miRNA regulation in the etiology of BPD, including Notch signaling (e.g., *JAG1*), axonal growth, and guidance (e.g., *CNTN4*, *NFIB*, *NAV3*, and *PTPRD*), and cholesterol homeostasis (e.g., *ABCA1*) (Hekimi and Kershaw, 1993; Bixby, 2000; Karasinska et al., 2009; Mason et al., 2009; Shimoda and Watanbbe, 2009; Pedroso et al., 2012). In addition, our identified BPD-associated pathways consisted of many collagen related genes, such as *COL1A2*, *COL27A1*, and *COL5A1*, implicating that these genes may augment their impacts on BPD through miRNA regulation. Although the connection of collagen and BPD was rarely reported in the literature, the emerging data and evidence, came from *in vivo* studies suggested that collagen joined the processes of axonal growth and guidance, synaptogenesis, and Schwann cell myelination during the development of nervous system (Hubert et al., 2009).

Among these identified miTGs (in Table 4), some of them have been previously linked to the regulation of miRNA machinery. A recent study showed the mediation of miR-34a and miR-21 on expression of *JAG1*, to regulate the differentiation of human monocyte-derived dendritic cell, which is involved in five of our identified curated gene sets (Hashimi et al., 2009). In addition, increased expression of *NAV3* mRNA was observed in brain tissue of Alzheimer’s disease and was suggested to be regulated by miR-29a (Shioya et al., 2010). On the contrary, the links between miRNAs and BPD were rarely constructed. Most of psychiatric- and neurological-associated miRNAs used in this study were reported to be related to schizophrenia, Alzheimer disease, autism, and Parkinson disease (according to PhenomiR database). Future studies are also needed to uncover the impacts of these miRNAs on the etiology of BPD.

There are some limitations in the present study. First, *P*_min_-value was used to represent the significance level of a gene. The information of other SNPs in a gene region may be missed. Nevertheless, previous studies showed that using *P*_min_-value in pathway analysis provides consistent results with other measures of gene-level statistic (Torkamani et al., 2008; Baranzini et al., 2009). Second, despite using more comprehensive pathway (e.g., MsigDB) and miRNA-disease/phenotype (e.g., PhenomiR) databases, the incompleteness of annotated pathways and miRNA-disease/phenotype information could have impacts on the correctness of the identified BPD-associated pathways. Third, the target genes of psychiatric- and neurological-associated miRNAs were predicted by computational methods. Although we used two miRNA target prediction algorithms to increase the correctness of prediction, further experimental validation by using functional studies are still needed in the future.

In conclusion, with integrating currently known psychiatric- and neurological-associated miRNAs as prior information, our pathway-based analyses using the GWA dataset of BPD identified not only previously reported pathways, but also new pathways that showed intriguing biological plausibility for BPD. So far, miRNAs studies in BPD are still in the infant stage. Our findings provided further evidence and support for exploring the roles of miRNA regulation in relation to nervous system for the risk of developing bipolar illness, especially ion channel regulation and axonal development. More, investigations remain to be done to elucidate the functions of these candidate pathways and genes, and the potential mechanisms involved with miRNA-mediated regulation.

## Conflict of Interest Statement

The authors declare that the research was conducted in the absence of any commercial or financial relationships that could be construed as a potential conflict of interest.

## Footnotes

^1^http://www.ncbi.nlm.nih.gov/gap

^2^http://www.microrna.org/microrna/home.do

## Appendix

**Table A1:** **Number of pathways at different cut-off in four statistical scenarios (total number of pathways = 4051)**.

Significance criterion	GSEA		SUM	
	Non-weighted	Weighted	Non-weighted	Weighted
Empirical *P*-value < 0.01	267	269	353	675
FDR_BH_ < 0.01	48	40	140	363

*GSEA, gene set enrichment analysis; SUM, sum-statistic approach; FDR_BH_, false discovery rate with control of multiple testing by using Benjamini & Hochberg’s method*.

**Table A2:** **Number of pathways identified by GSEA or SUM methods at significant level of 0.01 under weighted and non-weighted schemes (total number of pathways = 4051)**.

Non-weighted		Weighted			
		GSEA		SUM	
		≥0.01	<0.01	≥0.01	<0.01
Empirical *P*-value	≥0.01	3685	99	3374	324
	<0.01	97	170	2	351
FDR_BH_	≥0.01	3983	20	3688	223
	<0.01	28	20	0	140

*FDR_BH_, false discovery rate with control of multiple testing by using Benjamini & Hochberg’s method*.

**Table A3:** **Sixty-two enriched pathways at a FDR_BH_ < 0.01 level under weighted and non-weighted scenarios using GSEA and SUM methods**.

Pathway	Type	No. of genes in pathway	No. of miRNA target genes	No. of non-miRNA target genes	% of miRNA target genes*	GSEA				SUM			
						Non-weighted		Weighted		Non-weighted		Weighted	
						Empirical *P*-value	FDR_BH_	Empirical *P*-value	FDR_BH_	Empirical *P*-value	FDR_BH_	Empirical *P*-value	FDR_BH_
GO_CATION_TRANSMEMBRANE_ TRANSPORTER_ACTIVITY	GO	211	109	85	56.2	**<2e-4**	**<2e-4**	**<2e-4**	**<2e-4**	**<2e-4**	**<2e-4**	**<2e-4**	**<2e-4**
GO_GATED_CHANNEL_ACTIVITY	GO	121	55	59	48.2	**<2e-4**	**<2e-4**	**<2e-4**	**<2e-4**	**<2e-4**	**<2e-4**	**<2e-4**	**<2e-4**
GO_ION_TRANSMEMBRANE_ TRANSPORTER_ACTIVITY	GO	275	128	123	51.0	**<2e-4**	**<2e-4**	**<2e-4**	**<2e-4**	**<2e-4**	**<2e-4**	**<2e-4**	**<2e-4**
GO_NERVOUS_SYSTEM_DEVELOPMENT	GO	382	191	135	58.6	**<2e-4**	**<2e-4**	**<2e-4**	**<2e-4**	**<2e-4**	**<2e-4**	**<2e-4**	**<2e-4**
GO_SUBSTRATE_SPECIFIC_ TRANSMEMBRANE_TRANSPORTER_ACTIVITY	GO	341	151	157	49.0	**<2e-4**	**<2e-4**	**<2e-4**	0.014	**<2e-4**	**<2e-4**	**<2e-4**	**<2e-4**
GO_GTPASE_REGULATOR_ACTIVITY	GO	117	65	41	61.3	**<2e-4**	**<2e-4**	**<2e-4**	0.023	**<2e-4**	**<2e-4**	**<2e-4**	**<2e-4**
GO_POTASSIUM_CHANNEL_ACTIVITY	GO	50	30	19	61.2	**<2e-4**	**<2e-4**	**<2e-4**	0.023	**<2e-4**	**0.006**	**<2e-4**	**0.005**
GO_SUBSTRATE_SPECIFIC_ TRANSPORTER_ACTIVITY	GO	388	166	184	47.4	**<2e-4**	**<2e-4**	**0.001**	0.030	**<2e-4**	**<2e-4**	**<2e-4**	**<2e-4**
GO_VOLTAGE_GATED_POTASSIUM_ CHANNEL_COMPLEX	GO	40	21	17	55.3	**<2e-4**	0.013	**<2e-4**	0.014	**<2e-4**	**<2e-4**	**<2e-4**	**0.003**
GO_SYNAPTIC_TRANSMISSION	GO	172	77	81	48.7	**<2e-4**	0.021	**<2e-4**	**<2e-4**	**<2e-4**	**0.006**	**<2e-4**	**<2e-4**
GO_NEUROLOGICAL_SYSTEM_PROCESS	GO	377	139	202	40.8	**<2e-4**	0.021	**<2e-4**	**<2e-4**	**0.001**	0.021	**<2e-4**	**<2e-4**
GO_ION_CHANNEL_ACTIVITY	GO	147	70	69	50.4	**0.001**	0.036	**<2e-4**	**<2e-4**	**<2e-4**	**<2e-4**	**<2e-4**	**<2e-4**
GO_SUBSTRATE_SPECIFIC_CHANNEL_ ACTIVITY	GO	154	74	72	50.7	**0.001**	0.041	**<2e-4**	**<2e-4**	**<2e-4**	**<2e-4**	**<2e-4**	**<2e-4**
GO_TRANSMISSION_OF_NERVE_IMPULSE	GO	187	83	90	48.0	**0.001**	0.041	**<2e-4**	**<2e-4**	**0.001**	0.018	**<2e-4**	**<2e-4**
GO_NEUROGENESIS	GO	93	46	40	53.5	**<2e-4**	**<2e-4**	**0.003**	0.084	**<2e-4**	**0.006**	**<2e-4**	**<2e-4**
GO_GENERATION_OF_NEURONS	GO	83	42	34	55.3	**<2e-4**	**<2e-4**	**0.005**	0.109	**<2e-4**	**0.006**	**<2e-4**	**<2e-4**
GO_NEURON_DIFFERENTIATION	GO	76	38	31	55.1	**<2e-4**	**<2e-4**	0.011	0.153	**<2e-4**	**<2e-4**	**<2e-4**	**<2e-4**
GO_CYTOSKELETON	GO	361	173	150	53.6	0.030	0.285	**<2e-4**	**<2e-4**	0.042	0.252	**<2e-4**	**<2e-4**
KEGG_CALCIUM_SIGNALING_PATHWAY	KEGG	178	80	73	52.3	**<2e-4**	**<2e-4**	**<2e-4**	0.014	**<2e-4**	**<2e-4**	**<2e-4**	**<2e-4**
KEGG_ECM_RECEPTOR_INTERACTION	KEGG	84	48	32	60.0	**<2e-4**	**<2e-4**	0.011	0.153	**<2e-4**	**<2e-4**	**<2e-4**	**<2e-4**
KEGG_ARRHYTHMOGENIC_RIGHT_ VENTRICULAR_CARDIOMYOPAT_ARVC	KEGG	76	42	28	60.0	**<2e-4**	**<2e-4**	0.044	0.287	**<2e-4**	**<2e-4**	**<2e-4**	**<2e-4**
KEGG_FOCAL_ADHESION	KEGG	201	114	66	63.3	**<2e-4**	**<2e-4**	0.065	0.330	**<2e-4**	**<2e-4**	**<2e-4**	**<2e-4**
KEGG_NEUROACTIVE_LIGAND_RECEPTOR_ INTERACTION	KEGG	272	94	144	39.5	**<2e-4**	0.021	**<2e-4**	**<2e-4**	0.012	0.123	**<2e-4**	**<2e-4**
KEGG_RETINOL_METABOLISM	KEGG	64	19	38	33.3	**0.001**	0.029	**<2e-4**	**<2e-4**	0.032	0.215	**<2e-4**	**<2e-4**
KEGG_DRUG_METABOLISM_OTHER_ ENZYMES	KEGG	51	20	24	45.5	**0.002**	0.057	**<2e-4**	**<2e-4**	0.105	0.425	**<2e-4**	**<2e-4**
ACEVEDO_LIVER_CANCER_WITH_ H3K27ME3_UP	Curated	295	109	105	50.9	**<2e-4**	**<2e-4**	**<2e-4**	**<2e-4**	**<2e-4**	**<2e-4**	**<2e-4**	**<2e-4**
ACEVEDO_LIVER_CANCER_WITH_ H3K9ME3_UP	Curated	141	50	55	47.6	**<2e-4**	**<2e-4**	**<2e-4**	**<2e-4**	**<2e-4**	**<2e-4**	**<2e-4**	**<2e-4**
BERTUCCI_MEDULLARY_VS_DUCTAL_ BREAST_CANCER_DN	Curated	177	88	54	62.0	**<2e-4**	**<2e-4**	**<2e-4**	**<2e-4**	**<2e-4**	**<2e-4**	**<2e-4**	**<2e-4**
DACOSTA_UV_RESPONSE_VIA_ERCC3_ TTD_DN	Curated	76	50	19	72.5	**<2e-4**	**<2e-4**	**<2e-4**	**<2e-4**	**<2e-4**	**<2e-4**	**<2e-4**	**<2e-4**
HAMAI_APOPTOSIS_VIA_TRAIL_UP	Curated	334	176	134	56.8	**<2e-4**	**<2e-4**	**<2e-4**	**<2e-4**	**<2e-4**	**<2e-4**	**<2e-4**	**<2e-4**
LINDGREN_BLADDER_CANCER_CLUSTER_ 3_DN	Curated	223	98	86	53.3	**<2e-4**	**<2e-4**	**<2e-4**	**<2e-4**	**<2e−4**	**<2e-4**	**<2e-4**	**<2e-4**
MANALO_HYPOXIA_UP	Curated	211	123	58	68.0	**<2e-4**	**<2e-4**	**<2e-4**	**<2e-4**	**<2e-4**	**<2e-4**	**<2e-4**	**<2e-4**
MARTINEZ_RESPONSE_TO_TRABECTEDIN	Curated	42	26	13	66.7	**<2e-4**	**<2e-4**	**<2e-4**	**<2e-4**	**<2e-4**	**<2e-4**	**<2e-4**	**<2e-4**
RIGGI_EWING_SARCOMA_PROGENITOR_UP	Curated	429	222	133	62.5	**<2e-4**	**<2e-4**	**<2e-4**	**<2e-4**	**<2e-4**	**<2e-4**	**<2e-4**	**<2e-4**
SILIGAN_BOUND_BY_EWS_FLT1_FUSION	Curated	36	18	16	52.9	**<2e-4**	**<2e-4**	**<2e-4**	**<2e-4**	**<2e-4**	**<2e-4**	**<2e-4**	**<2e-4**
VECCHI_GASTRIC_CANCER_EARLY_DN	Curated	394	161	125	56.3	**<2e-4**	**<2e-4**	**<2e-4**	**<2e-4**	**<2e-4**	**<2e-4**	**<2e-4**	**<2e-4**
VERHAAK_AML_WITH_NPM1_MUTATED_DN	Curated	266	130	93	58.3	**<2e-4**	**<2e-4**	**<2e-4**	**<2e-4**	**<2e-4**	**<2e-4**	**<2e-4**	**<2e-4**
WANG_SMARCE1_TARGETS_UP	Curated	170	92	52	63.9	**<2e-4**	**<2e-4**	**<2e-4**	**<2e-4**	**<2e-4**	**<2e-4**	**<2e-4**	**<2e-4**
ONDER_CDH1_SIGNALING_VIA_CTNNB1	Curated	85	45	31	59.2	**<2e-4**	**<2e-4**	**<2e-4**	**<2e-4**	**<2e-4**	**<2e-4**	**<2e-4**	**0.003**
SABATES_COLORECTAL_ADENOMA_DN	Curated	292	118	112	51.3	**<2e-4**	**<2e-4**	**<2e-4**	**<2e-4**	**<2e-4**	**0.006**	**<2e-4**	**<2e-4**
HATADA_METHYLATED_IN_LUNG_CANCER_UP	Curated	367	161	144	52.8	**<2e-4**	**<2e-4**	**<2e-4**	**<2e-4**	**0.001**	0.014	**<2e-4**	**<2e-4**
ONDER_CDH1_TARGETS_2_UP	Curated	259	140	79	63.9	**<2e-4**	**<2e-4**	**<2e-4**	0.014	**<2e-4**	**<2e-4**	**<2e-4**	**<2e-4**
STARK_PREFRONTAL_CORTEX_22Q11_ DELETION_UP	Curated	212	122	40	75.3	**<2e-4**	**<2e-4**	**<2e-4**	0.014	**<2e-4**	**<2e-4**	**<2e-4**	**<2e-4**
JAATINEN_HEMATOPOIETIC_STEM_CELL_UP	Curated	325	155	110	58.5	**<2e-4**	**<2e-4**	**0.001**	0.042	**<2e-4**	**<2e-4**	**<2e-4**	**<2e-4**
KINSEY_TARGETS_OF_EWSR1_FLII_ FUSION_DN	Curated	336	166	98	62.9	**<2e-4**	**<2e-4**	**0.001**	0.042	**<2e-4**	**<2e-4**	**<2e-4**	**<2e-4**
BROWNE_HCMV_INFECTION_24HR_DN	Curated	153	69	63	52.3	**<2e-4**	**<2e-4**	**0.001**	0.046	**<2e-4**	**<2e-4**	**<2e-4**	**<2e-4**
DELYS_THYROID_CANCER_DN	Curated	214	109	86	55.9	**<2e-4**	0.013	**<2e-4**	**<2e-4**	**<2e-4**	**<2e-4**	**<2e-4**	**<2e-4**
HORIUCHI_WTAP_TARGETS_UP	Curated	323	147	119	55.3	**<2e-4**	0.013	**<2e-4**	**<2e-4**	**<2e-4**	**<2e-4**	**<2e-4**	**<2e-4**
THUM_SYSTOLIC_HEART_FAILURE_DN	Curated	248	136	61	69.0	**0.001**	0.029	**<2e-4**	**<2e-4**	**<2e-4**	**<2e-4**	**<2e-4**	**<2e-4**
IWANAGA_CARCINOGENESIS_BY_KRAS_ PTEN_DN	Curated	445	161	119	57.5	**0.001**	0.036	**<2e-4**	**<2e-4**	**<2e-4**	**<2e-4**	**<2e-4**	**<2e-4**
SCHUETZ_BREAST_CANCER_DUCTAL_ INVASIVE_UP	Curated	364	172	146	54.1	**<2e-4**	**<2e-4**	**0.001**	0.051	**<2e-4**	**<2e-4**	**<2e-4**	**<2e-4**
GRESHOCK_CANCER_COPY_NUMBER_DN	Curated	347	198	107	64.9	**<2e-4**	**<2e-4**	**0.002**	0.055	**<2e-4**	**<2e-4**	**<2e-4**	**<2e-4**
CHEBOTAEV_GR_TARGETS_DN	Curated	140	64	42	60.4	**<2e-4**	**<2e-4**	**0.003**	0.077	**<2e-4**	**<2e-4**	**<2e-4**	**<2e-4**
GRESHOCK_CANCER_COPY_NUMBER_UP	Curated	322	195	107	64.6	**<2e-4**	**<2e-4**	**0.003**	0.084	**<2e-4**	**<2e-4**	**<2e-4**	**<2e-4**
ODONNELL_METASTASIS_UP	Curated	84	35	31	53.0	**<2e-4**	**<2e-4**	**0.004**	0.093	**<2e-4**	**<2e-4**	**<2e-4**	**<2e-4**
SENESE_HDAC1_TARGETS_DN	Curated	267	105	96	52.2	**<2e-4**	**<2e-4**	**0.004**	0.096	**<2e-4**	**<2e-4**	**<2e-4**	**<2e-4**
DAVICIONI_MOLECULAR_ARMS_VS_ ERMS_UP	Curated	339	179	113	61.3	**<2e-4**	**<2e-4**	0.015	0.176	**<2e-4**	**<2e-4**	**<2e-4**	**<2e-4**
DOANE_RESPONSE_TO_ANDROGEN_DN	Curated	248	119	94	55.9	**0.006**	0.116	**<2e-4**	**<2e-4**	**0.001**	0.021	**<2e-4**	**<2e-4**
FOSTER_INFLAMMATORY_RESPONSE_ LPS_DN	Curated	486	219	122	64.2	0.017	0.205	**<2e-4**	**<2e-4**	**<2e-4**	**0.006**	**<2e-4**	**<2e-4**
ACEVEDO_LIVER_CANCER_WITH_ H3K27ME3_DN	Curated	226	73	87	45.6	0.019	0.216	**<2e-4**	**<2e-4**	0.210	0.615	**<2e-4**	**<2e-4**
BOYLAN_MULTIPLE_MYELOMA_C_D_DN	Curated	328	100	113	46.9	0.020	0.224	**<2e-4**	**<2e-4**	0.027	0.196	**0.001**	**0.009**
YAUCH_HEDGEHOG_SIGNALING_ PARACRINE_DN	Curated	385	104	108	49.1	0.058	0.396	**<2e-4**	**<2e-4**	**<2e-4**	**0.006**	**<2e-4**	**<2e-4**

**Proportion of miRNA target genes was obtained by calculating the number of miRNA target genes divided by the total number of miRNA target genes plus non-miRNA target genes*.

*Empirical *P*-value or FDR_BH_ with values less than 0.01 were shown in bold*.

*GO, Gene Ontology; KEGG, Kyoto Encyclopedia of Genes and Genomes; GSEA, gene set enrichment analysis; SUM, sum-statistic approach*.

*FDR_BH_, correct for false discovery rates using Benjamini and Hochberg’s method*.

*Empirical *P*-value and FDR_BH_ “<2e−4” meant that no one had greater score than the actual score among the 5,000 permutations for the analysis of each pathway*.
